# Metabolic Monitoring for Adults Living with a Serious Mental Illness on a Second-Generation Antipsychotic Agent: A Scoping Review

**DOI:** 10.1007/s10488-024-01408-9

**Published:** 2024-08-17

**Authors:** Tien Ngoc Thi Bui, Ruby Tszwai Au, Jack Luke Janetzki, Sara S. McMillan, Elizabeth Hotham, Vijayaprakash Suppiah

**Affiliations:** 1 Clinical and Health Sciences, University of South Australia, Adelaide, SA Australia; 2 Centre for Mental Health, Griffith University, Brisbane, QLD Australia; 3 Menzies Health Institute Queensland, Griffith University, Gold Cost, QLD Australia; 4 School of Pharmacy and Medical Sciences, Griffith University, Gold Coast, QLD Australia; 5 Australian Centre for Precision Health, University of South Australia, Adelaide, SA Australia

**Keywords:** Antipsychotic agents, Metabolic diseases, Monitoring, Mental illness, Healthcare

## Abstract

Premature mortality in people living with a severe mental illness (SMI) is often attributed to multiple factors including the use of medicines such as antipsychotics. Second-generation antipsychotics (SGAs) are known to cause metabolic syndrome which can increase the risk of cardiovascular disease. Practice guidelines have recommended regular physical health monitoring, particularly of metabolic parameters, however, metabolic monitoring for people living with SMI using antipsychotics remains suboptimal. Therefore, highlighting the need for ongoing research. This scoping review aimed to provide an overview of current metabolic monitoring practices. We anticipate that this information will assist clinicians and policymakers and inform future research. The following databases were searched: MEDLINE (Ovid), Embase (Ovid), CINAHL (EBSCO), the Cochrane Database of Systemic Reviews (Wiley), APA PsycInfo (Ovid) and Scopus (Elsevier Science Publishers). The target group was adults (aged ≥ 18) diagnosed with SMI (including bipolar disorder, major depressive disorder and psychotic disorders) and taking SGAs. In total, 44 studies from 14 countries were retrieved. Our findings highlighted that most studies conducted in hospitals did not report on metabolic monitoring practices. Additionally, the roles and responsibilities of healthcare professionals in metabolic monitoring for SMI were infrequently described and parameters such as waist circumference and BMI were often poorly monitored. The scoping review highlights that no streamlined approach towards metabolic monitoring currently exists. There is a need to stipulate and define the roles and responsibilities of all health professionals involved in metabolic monitoring in SMI to optimise care for these individuals. Moreover, there is a need for ongoing research, particularly in the community setting, to promote increased accessibility to metabolic monitoring for SMI.

**Supplementary Information:**

The online version contains supplementary material available at 10.1007/s10488-024-01408-9.

## Background

Reports indicate that people living with a severe mental illness (SMI) have a reduced life expectancy compared to the general population, with estimates ranging from 10 to 20 years (Liu et al., 2017). Premature mortality has been linked to various factors, including cardiovascular disease (CVD) (Lawrence et al., 2010). Research indicates that individuals with SMI are more likely to report higher rates of tobacco smoking, poorer diet and low physical activity (Wichniak et al., 2019), all of which contribute to an elevated risk of CVD (Scott & Happell, 2011). Furthermore, commonly prescribed medications such as antipsychotics can amplify this risk by increasing the likelihood of developing metabolic syndrome (MetSyn) (Dekker et al., 2005), which is characterised by elevated blood glucose, lipids and blood pressure, as well as central obesity (Penninx & Lange, 2018). Antipsychotics are categorised as either first-generation antipsychotics (FGAs) or second-generation antipsychotics (SGAs) based on their pharmacological properties. While SGAs offer improved tolerance due to a lower risk of extrapyramidal side effects than FGAs (D’Souza & Hooten, 2021), they introduce a heightened risk of metabolic disturbances, including MetSyn (Bernardo et al., 2021; Hasan et al., 2013; Dekker et al., 2005).

Global consensus or treatment guidelines (American Diabetes Association, 2004; Castle et al., 2017) stipulate the need for regular physical health monitoring for patients with SMIs taking antipsychotics. The American guidelines recommend monitoring metabolic parameters (including weight, waist circumference, blood pressure, plasma glucose and lipid profile) at baseline and at 4, 8 and 12 weeks and annually thereafter as part of routine care (American Diabetes Association, 2004). Similarly, Australian guidelines recommend regular monitoring of metabolic parameters, with an additional review at 24 weeks after antipsychotic initiation (Castle et al., 2017).

Previous research has shown suboptimal metabolic monitoring rates in patients with SMI (Chee et al., 2017; Cohn & Sernyak, 2006; Michael & MacDonald, 2020), highlighting a disparity between guidelines and existing practices (Cunningham et al., 2018; Mackin et al., 2007; Mead et al., 2021). A multi-country systematic review and meta-analysis of 218,940 patients (inpatient and community-dwelling) reported that only blood pressure and triglycerides were routinely monitored for at least 50% of participants, while weight (47.9%), blood glucose (44.3%) and cholesterol (41.5%) were measured in fewer than half of the study cohort (Mitchell et al., 2012). Suboptimal monitoring rates were also identified in one Australian inpatient ward (n = 61), where height and weight were measured in less than half (46%) of the patients, while lipid levels were measured 23% of the time (Michael & MacDonald, 2020). However, this single-site study had a small sample size and may not reflect procedures in other practice settings.

Several studies have explored the role of allied health professionals, such as nurses (Chee et al., 2017) and pharmacists (Al AdAwi et al., 2020; Sud et al., 2021) in the management of cardiometabolic risk, metabolic syndrome (MetSyn) and related diseases in the SMI. A systematic review revealed that interventional studies to improve metabolic monitoring rates in patients with SMI generated relatively positive results (Melamed et al., 2019). These aforementioned studies focused on quantifying and improving metabolic monitoring rates but did not provide details on practice implementation. For example, a systematic review by Mitchell and colleagues quantified and compared the rates of metabolic monitoring before and after guideline implementation (Mitchell et al., 2012) but did not describe the processes of metabolic monitoring. Reviews to date have not reported on the context of interventions; for example, who are the health professionals involved in metabolic monitoring, where metabolic monitoring commonly occurs (for example, primary compared to tertiary settings) and how (such as procedures and systems) is this monitoring integrated and active in practice (Melamed et al., 2019; Poojari et al., 2022)? Addressing these questions will inform future implementations of more targeted and streamlined interventional approaches.

It is important to understand current metabolic monitoring patterns in routine clinical practice to improve the management and care of SMI. This scoping review provides an overview of current metabolic monitoring practices by utilising published studies’ descriptions of existing baseline monitoring rates and procedures (that is, without the influence of study interventions) as proxy measures. This review summarised and mapped existing metabolic monitoring practices, highlighted current gaps in practice and provided directions for future research initiatives. We anticipate that this information will assist clinicians and policymakers and inform future research.

## Methods

The scoping review followed the Preferred Reporting Items for Systematic Reviews and Meta-Analyses extension for Scoping Reviews (PRISMA-ScR) checklist (Tricco et al., 2018) and the Joanna Briggs Institute (JBI) Manual for Evidence Synthesis updated methodological guidance for the conduct of scoping reviews (Peters et al., 2021). An a priori protocol was developed and registered on the Open Science Framework (https://doi.org/10.17605/OSF.IO/YMR5C).

An initial limited search of MEDLINE (Ovid) and APA PsycInfo (Ovid) was performed. Keywords in the titles and abstracts of relevant articles were used to develop the full search strategy (Supplementary Information). The search strategies, inclusion/exclusion criteria and the data extraction tool were piloted by two members of the research team on a small sample (n = 8) of papers. The following databases were searched: MEDLINE (Ovid), Embase (Ovid), CINAHL (EBSCO), the Cochrane Database of Systematic Reviews (Wiley), APA PsycInfo (Ovid) and Scopus (Elsevier Science Publishers). The search was adapted for each database and information source. Regular input from an academic librarian further refined the search strategy and translation across different databases. A search for grey literature was undertaken via Google Scholar and ProQuest Dissertation and Theses. The scoping review was informed by the Population, Concept, Context framework (Pollock et al., 2023). The search was conducted on the 8th of September 2022 and updated on the 13th of October 2023.

### Population

The target group was adults (aged ≥ 18) diagnosed with SMI (including bipolar disorder, major depressive disorder and psychotic disorders) and taking SGAs.

### Concept

This scoping review examined the combination of the following concepts:

1. SMI was diagnosed in adults with the following conditions: bipolar disorder, major depressive disorder and psychotic disorders (including schizophrenia and schizoaffective disorder)
2. Currently taking SGAs
3. The metabolic syndrome incidence, cardiometabolic risk and metabolic parameters included the following:
  - Weight
  - Waist circumference
  - Blood pressure (BP)
  - Plasma glucose levels, such as blood glucose levels (BGLs) and haemoglobin A1c (HbA1c) levels
  - Lipid levels included total cholesterol (TC), high-density lipoprotein (HDL), low-density lipoprotein (LDL) and triglyceride (TG) levels.
4. Processes of metabolic monitoring (such as onsite or referral for laboratory tests)

### Context

The scoping review included studies conducted in healthcare facilities such as community settings, medical centres, hospitals, and specialised care facilities. There were no attempts to limit the search to specific countries.

### Inclusion and Exclusion Criteria

This review considered a variety of study designs, including qualitative, experimental, quasi-experimental, “before and after”, analytical observational, retrospective, cross-sectional and descriptive observational studies. Studies that monitored at least three metabolic parameters (weight, blood pressure, waist circumference, plasma glucose and lipid levels) and were published after 2004 (aligning with the publication date of the American guideline (American Diabetes Association, 2004)) were included.

Studies that were deemed not to reflect real-life practice, such as randomised controlled trials were excluded. Prospective studies were also excluded as baseline monitoring was often conducted as part of the research methodology and therefore may not reflect the usual metabolic monitoring rates in the particular setting. Additionally, case–control reports, case studies or case series were not considered because they cannot be generalised to the broader cohort of SMIs. Finally, studies that were not published in English or for which the full text could not be retrieved (for example, through interlibrary loans) were excluded.

### Data Screening and Extraction

The articles retrieved by the search were managed by EndNote X9 (Clarivate Analytics, PA, USA), and Covidence (Veritas Health Innovation, Melbourne, Australia) was used to screen the studies. The titles and abstracts of the identified studies were screened by two independent reviewers, and conflicts were resolved by a third reviewer. An overly inclusive approach was employed, with full-text articles obtained for any abstracts in doubt. All studies meeting the inclusion criteria were retrieved in full and underwent the same screening process as the first phase (described above). Data extraction was conducted by two researchers, who extracted data from half of the studies and cross-verified the other half. The extracted information included author, year of publication, country, sample size, study design, role and responsibilities of health care professionals, setting, monitoring process and outcomes measured. The reference lists of relevant systematic reviews were screened independently by two authors for potentially relevant studies. The findings of the review are reported using descriptive analysis.

### Data Analysis

Relevant data from the retrieved studies were charted and organised into the data extraction table. Additional information was collated and summarised (Arksey & O’Malley, 2005) then presented as appropriate tables or figures. The type and frequency of metabolic monitoring for the relevant metabolic parameters, including HDL and TG when reported, was included in a graph to illustrate the range of metabolic monitoring rates across the included studies.

## Results

### Study Selection

**Fig. 1:**
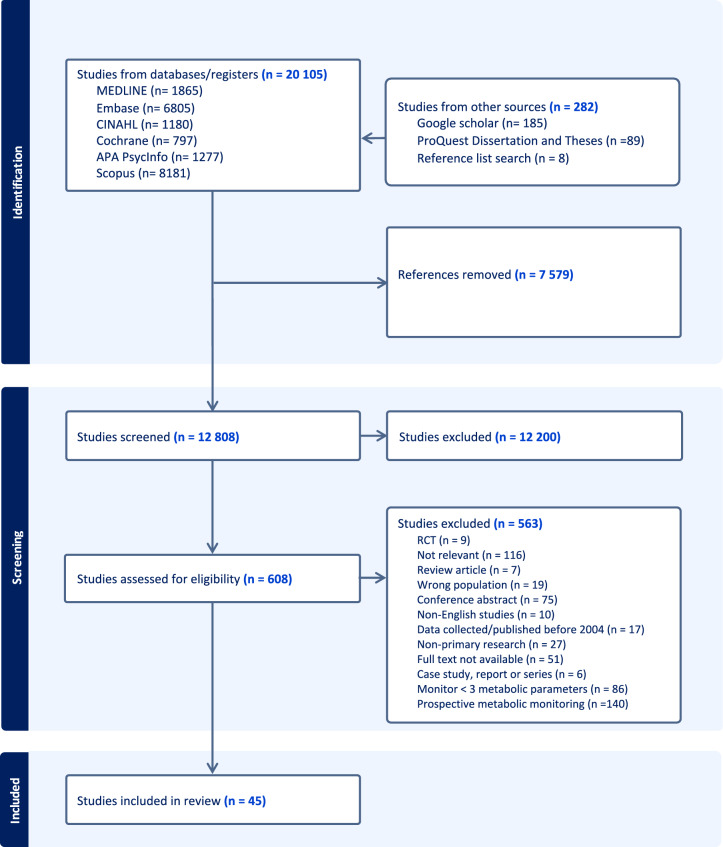
PRISMA flow diagram showing the process of study selection for inclusion in the scoping review

Of the 20,387 studies identified from the initial search, 7579 duplicates were removed using Endnote and Covidence. The first screening phase identified 608 studies for full-text review (Fig. 1). Systematic and literature reviews (n = 3) were assessed, and an additional two studies were identified. A study that included a small cohort of children was also included, as the majority of the participants were adults (Cotes et al., 2015).

### Study Characteristics

In total, 45 studies were included (Fig. 1), and two manuscripts (a thesis and a peer review) published by the same authors covering the same content were considered single inclusions. The 44 included studies were conducted in the following countries (Table 1): the UK (n = 13) (Ali et al., 2023; Barnes et al., 2007, 2008, 2015, 2020; Gumber et al., 2010; Harrison et al., 2012; Holt et al., 2010; Lau et al., 2019; Mwebe et al., 2020; Najim & Islam, 2013; Pearsall et al., 2019; Ross et al., 2018), the US (n = 9) (Batscha et al., 2010; Butler et al., 2013; Coakley et al., 2012; Cotes et al., 2015; Kilbourne et al., 2007; Kioko et al., 2016; Mittal et al., 2014; Pereira et al., 2019; Tatreau et al., 2016), Ireland (n = 4) (Feeney & Mooney, 2005; Kelly et al., 2022; Lydon et al., 2021; O’Callaghan et al., 2011), Australia (n = 3) (Nguyen et al., 2009; Thompson et al., 2011; Viglione & Short, 2021), New Zealand (n = 2) (Keenan et al., 2020; O’Brien & Abraham, 2021), Canada (n = 2) (Fontaine et al., 2022; Stephenson et al., 2023), Denmark (n = 2) (Kjeldsen et al., 2013; Knudsen et al., 2022), South Africa (n = 2) (Marsay & Szabo, 2010; Shamima Saloojee et al., 2014a, 2014b) and one each from France (Verdoux et al., 2008), Germany (Deuschle et al., 2013), Malaysia (Hor et al., 2016), Singapore (Tan et al., 2022), South India (Poojari et al., 2020), and Spain (Bobes et al., 2011). One did not specify the country in which the study was conducted (Bomboy et al., 2021). More than half of the studies were conducted within the last 10 years (n = 29) (Ali et al., 2023; Barnes et al., 2015; Barnes et al., 2020; Bomboy et al., 2021; Butler et al., 2013; Cotes et al., 2015; Deuschle et al., 2013; Fontaine et al., 2022; Hor et al., 2016; Keenan et al., 2020; Kelly et al., 2022; Kioko et al., 2016; Kjeldsen et al., 2013; Knudsen et al., 2022; Lau et al., 2019; Lydon et al., 2021; Mittal et al., 2014; Mwebe et al., 2020; Najim & Islam, 2013; O’Brien & Abraham, 2021; Pearsall et al., 2019; Pereira et al., 2019; Poojari et al., 2020; Ross et al., 2018; Shamima Saloojee et al., 2014a, 2014b; Stephenson et al., 2023; Tan et al., 2022; Tatreau et al., 2016; Viglione & Short, 2021).

**Table 1:** Data extraction table, presented in order of publication year

Author(s), country	Study type	Setting, sample size	Role and responsibilities of health care professional	Specific monitoring process reported	Outcome measure* Results for initial audit (preintervention) only
Ali et al., 2023, UK (Ali et al., 2023)	Observational retrospective study	Primary care, n = 479 records	Not reported	Not reported	*Less than 1 monitoring over 3 years* BP: 24.55% BMI: 30.99% HDL: 39.03% Non-HDL: 38.63% HbA1c: 1.01% Waist Circumference: 3.42% *One monitoring in three years* BP: 16.7% BMI: 21.33% HDL: 14.69% Non-HDL: 14.08% HbA1c: 0% Waist Circumference: 0.4% *More than one monitoring in three years* BP: 49.9% BMI: 34.41% HDL: 16.1% Non-HDL: 16.1% HbA1c: 0% Waist Circumference: 0%
Stephenson et al., 2023, Canada (Stephenson et al., 2023)	Cohort study	Primary care, n = 2643 records	Family physician	Not reported	*With one or more measurements* Pre-pandemic: BP: 58.95% LDL: 42.98% HbA1C: 51.15% Pandemic: BP: 27.64% LDL: 32.22% HbA1c: 39.82%
Knudsen et al., 2022, Denmark (Knudsen et al., 2022)	Cross-sectional study	Psychiatric outpatient clinics, n = 107	Not reported	Not reported	Participants with a measurement in the past year for: BP: 80.4% Cholesterol: 92.5% HbA1c: 92.5%
Fontaine et al., 2022, Canada (Fontaine et al., 2022)	Cross sectional study	Tertiary hospital, n = 402	Nurses BP and weight/BMI measurements were routine nursing practice in this psychiatry unit	Not reported	BP: 99.8% Weight/BMI: 97.8% Lipid profile: 24.4% Fasting glucose/HbA1c: 33.3% Waist circumference: 4.5% All monitored: 1.24%
Kelly et al., 2022, Ireland (Kelly et al., 2022)	Pre/post intervention study	Hospital First Episode Psychosis n = 33 First Episode Psychosis reaudit n = 20 Chronic psychosis n = 41	Not reported	Not reported	Baseline for First Episode Psychosis: BP: 88% Weight: 67% BMI: 3% Fasting lipids: 61% Fasting glucose: 39% HbA1c: 0% Waist circumference: 3% Heart rate: 91% Prolactin: 18% ECG: 67% Chronic psychosis: BP: 20% Weight: 17% BMI: 9.7% Fasting lipids: 17% Fasting glucose: 17% Waist circumference: 0% Diabetes (as reported): 4%
Tan et al., 2022, Singapore (Tan et al., 2022)	Retrospective cohort study	Hospital, n = 5256	Not reported	Not reported	During inpatient period BP: 24.5% BMI: 5.6% HDL: 16.8% LDL: 16.1% Fasting glucose: 14.7% HbA1c: 7.3% During recommended period of CVD risk factor management BP: 85.8% BMI: 45.7% HDL: 18.2% LDL: 18.2% Fasting glucose: 16.3% HbA1c: 15.4%
Bomboy et al., 2021, country not specified (Bomboy et al., 2021)	Pre/post intervention study with control group	Rural community mental health centre Intervention group n = 82 pre implementation of intervention n = 91 post implementation Control group n = 129 pre intervention period n = 135 post intervention period	Psychiatric prescribers (Psychiatrists and advanced practice nurses) Determine if laboratory work was needed and provided verbal order for laboratory tests Clinic Staff (Medical assistant or certified nursing assistant) Laboratory work	Patients were identified by medication clinic staff and assessed/reviewed by prescriber. Samples for laboratory tests were drawn onsite, specimens transported and processed at the main laboratory site and results were uploaded to the portal for the medication clinic staff to access and record on the metabolic monitoring form. Prescriber determined if laboratory work was needed, provided verbal order for medication clinic staff (to do lab work and document).	Intervention group Laboratory tests ordered: 1 out of 82 (1.2%) Control group Laboratory tests: 5 out of 129 (3.8%)
Lydon et al., 2021, Ireland (Lydon et al., 2021)	Cross sectional study	Mental Health Services at University Hospital Clozapine group n = 119 Long-acting injection (LAI) – antipsychotic group n = 117	Not reported	All laboratory data examined were analysed at the biochemistry laboratory at the University Hospital. Dedicated clozapine clinic staffed by clinical nurse specialists. Manuscript did not report who measured the metabolic parameters.	Clozapine cohort BP: 100% Weight: 96% Cholesterol: 95% HDL: 95% LDL: 90% Triglycerides: 95% Any glucose measure: 95% Waist circumference: 2% LAI antipsychotic cohort BP: 39% Weight: 23% Cholesterol: 95% HDL: 95% LDL: 90% Triglycerides: 95% Any glucose measure: 95% Waist circumference: 15%
O’Brien & Abraham, 2021, New Zealand (O’Brien & Abraham, 2021)	Audit	Secondary mental health services and primary care Primary care audit n = 46 Secondary care audit n = 47 Practice nurse survey n = 24	Not reported	Not reported	Primary care: BP: 71.7% Weight: 58.7% HDL: 50% Triglycerides: 50% Fasting blood glucose: 0% Blood glucose: 63% Waist circumference: 6.5% Secondary services BP: 80.9% Weight: 76.6% HDL: 51% Triglycerides: 51% Fasting blood glucose: 0% Blood glucose HbA1c: 51% Waist circumference: 12.5%
Viglione & Short, 2021, Australia (Viglione & Short, 2021)	Pre/post intervention audit	Mental health inpatient service Preintervention n = 106	Nursing staff Measures physical parameters Junior medical officer Responsible for “bloodwork”	Not reported	BP: 99.1% BMI: 83% Fasting lipid profile: 20.8% HbA1c: 21.7% Waist circumference: 36.8%
Barnes et al., 2020, UK (Barnes et al., 2020)	Audit	UK member trusts and healthcare services as part of the Prescribing Observatory for Mental Health (n = 64) n = 6948 patients	Reported that data were collected by clinicians and clinical audit staff. * *Unclear if this refers to physical collection of metabolic parameters or review of case notes to collect data for the audit	Not reported	Pre-treatment screening BP: 97% Body weight: 83% Lipid levels: 81% Plasma glucose or HbA1c: 80% General physical examination conducted: 79% Monitoring in first 2 weeks of clozapine BP: 11% Patients treated with clozapine for more than 1 year (n = 5908) BP: 85% Body weight/BMI: 81% Plasma lipids: 73% Plasma glucose: 78% Physical examination: 55% 7% did not have any physical health checks documented in the clinical records in the previous year
Keenan et al., 2020, New Zealand (Keenan et al., 2020)	Audit	General practices Patients n = 117 General practices n = 8	Not reported	Not reported	BP: 85% Weight: 82% Lipid: 66% HbA1c: 70% Waist Circumference: 3% ECG: 9% Prolactin: 2% Complete Blood Count: 68%
Mwebe et al., 2020, UK (Mwebe et al., 2020)	Audit	Inpatient psychiatric wards, n = 120	Nurses Reports that "*nursing enquiries and discussions with patients at baseline or during patient stay in relation to unhealthy lifestyle behaviours were often succinct or missing*.”	Not reported	See Table 2
Poojari et al., 2020, South India (Poojari et al., 2020)	Retrospective cohort study	Tertiary care health institution with specialty psychiatry inpatient and outpatient clinics, n = 315	Not reported	Not reported	See Table 2
Lau et al., 2019, UK (Lau et al., 2019)	Audit	General practices, n = 57	Not reported	General practitioner, roles and responsibilities pertaining to metabolic monitoring not specified	BP: 80.70% Weight: 70.20% Blood lipids: 52.60% Fasting blood glucose: 31.60% HbA1c: 49.1% Waist circumference: 17.50% Pulse: 47.30% Prolactin: 7.00% Full blood count: 43.90% Urea and electrolytes: 66.70% Liver function tests: 52.60% Lifestyle advice: 38.60%
Pearsall et al., 2019, UK (Pearsall et al., 2019)	Cross sectional study	Community and inpatients- adult mental health services, n = 7718	Not reported	Not reported	One blood test in the preceding 2 years Cholesterol: 25.17% Triglycerides: 25.30% Glucose: 20.99% HbA1c: 13.66% Albumin: 12.54% Creatinine: 16.18% Alanine transaminase: 17.91% Two blood tests in the preceding 2 years Cholesterol: 24.60% Triglycerides: 24.48% Glucose: 21.66% HbA1c: 6.31% Albumin: 14.73% Creatinine: 15.83% Alanine transaminase: 17.54% Three or more blood tests in the preceding 2 years Cholesterol: 27.47% Triglycerides: 27.40% Glucose: 40.48% HbA1c: 11.13% Albumin: 57.06% Creatinine: 53.25% Alanine transaminase: 48.69%
Pereira et al., 2019, US (Pereira et al., 2019)	Audit (Chart review)	Outpatient psychiatric clinic, n = 54	Practitioners* Responsible for ordering tests Psychiatry clinical staff Involved in patient care * Not specified	Not reported	BP Baseline: 81% 12 weeks: 45% Annually: 70% Weight and BMI Baseline: 83% 4 weeks: 43% 8 weeks: 33% 12 weeks: 50% Quarterly: 49% Lipid Baseline: 42% Quarterly: 14% 5 years: Not reported Fasting blood glucose/haemoglobin A1c Baseline: 83% 12 weeks: 31% Annually: 82% Waist circumference Baseline: 0% Annually: 0%
Ross et al., 2018, UK (Ross et al., 2018)	Pre/post intervention audit	Secondary care setting Baseline audit (2012) n = 96	No specification of who ordered test, although stipulated that patient care is provided by consultant psychiatrists, psychiatry residents, nurses, occupational therapists and social workers. Family physician hospitalist (for any physical health concerns). Suggested that attending physician could order laboratory tests but not explicitly stated.	Medical directive All patients admitted under the inpatient psychiatry ward were automatically under investigations of height, weight, BMI, waist circumference, daily vitals for 3 days, blood tests including CBC, liver transaminases, kidney function tests, TSH, serum glucose and lipids after 72 h of admission. Glycosylated haemoglobin measured if the patient is known to be diabetic. Patients who have undergone the test within 6 months and were reported normal would not have them repeated automatically unless there was a clear indication. Attending physician could repeat these tests at any time.	BP: 92% Height and weight:75% Lipids: 36% Fasting or random glucose: 10% Blood glucose: 31% Waist circumference: 0%
Hor et al., 2016, Malaysia (Hor et al., 2016)	Pre/post intervention study	General public hospital, n = 300	Monitoring conducted by nurses and health care assistants	Not reported	Less than 10% of patients had their fasting blood glucose, fasting triglyceride, fasting HDL, height, weight, and waist circumference measured. Less than 20% had their BP measured.
Kioko et al., 2016, US (Kioko et al., 2016)	Pre/post intervention study	Outpatient mental health facility, n = 50 charts reviewed	Mental health clinicians/providers* are responsible in ordering blood work, screening and using the monitoring tool *Did not state whether there is a difference between mental health clinicians and providers	Not reported	69% laboratory tests not ordered. 22% laboratory tests done. 10% laboratory tests not done. Parameters measured: BP, weight, height, lipid panel, fasting glucose and/or glycated haemoglobin parameters
Tatreau et al., 2016, US (Tatreau et al., 2016)	Cross sectional study	Psychiatric inpatient units at the University of North Carolina Health Care System Unit A (reverse colocated medical care (RCL)) n = 220 Unit B (treatment as usual (TAU)) n = 232	Refer to monitoring process column	Unit A Laboratory values obtained by physician’s assistant supervised by a family physician. Unit B Medical care provided by resident psychiatrists supervised by attending psychiatrists. Hospitalists available for medical consultation. Standard admission orders include a basic chemistry panel, complete blood count, thyroid-stimulating hormone analysis, urinalysis, and urine toxicology screen. Laboratory tests ordered, completed, and reviewed prior to all admissions.	Unit A BP: 100% BMI: 49% Lipid:61% Glucose: 99% HbA1c: 56% Unit B BP: 100% BMI: 47% Lipid: 20% Glucose: 66% HbA1c: 16%
Barnes et al., 2015, UK (Barnes et al., 2015)	Pre/post intervention audit	Adult, assertive outreach, community psychiatric services in the UK (Multiple sites) People prescribed continuing antipsychotic medication under the care of assertive outreach community psychiatric services. Baseline audit (2006) n = 1966	Patients were treated by Assertive Outreach Teams* * Not specified	Patients were treated by Assertive outreach teams, however the specific monitoring process was not reported	Baseline/Preintervention (2006) No evidence of MetSyn screening: 46% Some evidence of MetSyn screening (mention of review of any of the four aspects of the MetSyn and/or documentation of up to three relevant test results): 43% Test result documented for all four aspects of MetSyn: 11%
Cotes et al., 2015, US (Cotes et al., 2015)	Pre/post intervention audit	Ten community mental health centres 2009—193 Adult, 37 children 2010—203 Adult, 32 children	Psychiatric prescribers- role and responsibilities not clearly described	Not reported	Baseline year (2009) BP recorded: 33% Past-year weight recorded: 52% Past-year cholesterol testing: 32% Triglyceride testing: 32% Glucose testing: 45% Abdominal girth recorded: 7%
Saloojee et al., 2014a, 2014b, South Africa (Shamima Saloojee et al., 2014a, 2014b)	Cross sectional study	General hospital -Psychiatric unit, n = 331	Not reported	Not reported	BP: 99% Fasting serum lipids: 1.8% Fasting blood glucose: 3.9% Random blood glucose: 96.6% Waist circumference: 0.6% All components: 0.6%
Mittal et al., 2014, US (Mittal et al., 2014)	Cohort study	Veterans Affairs medical centres Veterans n = 12,009	Not reported	Not reported	Baseline Weight: 66.6% Low-density lipoprotein: 32.1% Glucose or HbA1c: 45.8% 3 months follow-up Weight: 49.5% Low-density lipoprotein: 16.2% Glucose or HbA1c: 27.1%
Deuschle et al., 2013, Germany (Deuschle et al., 2013)	Multicentre cross-sectional study	In- and outpatient settings Hospitals n = 49 Patients n = 674	Refer to monitoring process column	Psychiatrists documented weight, height, waist circumference, total, LDL- and HDL-cholesterol, triglycerides, fasting glucose, HbA1c, and systolic and diastolic blood pressures. All data were derived from clinical routine.	BP: 37% BMI (weight and height): 54% Cholesterol: 25% HDL-Cholesterol: 8% LDL-Cholesterol: 8% Triglycerides: 25% Fasting glucose: 19% Waist circumference: 23%
Kjeldsen et al., 2013, Denmark (Kjeldsen et al., 2013)	Pre/post intervention study Two groups—Passive dissemination (PD group) and Active dissemination (AD group)	Psychiatric ward, University Hospital Implementation of guideline by passive dissemination group n = 93 Implementation of guideline by active implementation group n = 112	Nurses and physiotherapists Perform the screening Clinical Pharmacist Outreach visits	All metabolic laboratory measures were performed at one central laboratory. Outreach visits (intervention) were performed by experienced clinical pharmacists; and screening was performed by other staff, e.g. nurses or physiotherapists.	PD group Screening sheet was used for 36% of the patients. 22% patients had all five screening measurements documented in their medical charts. Waist circumference: 74% Fasting glucose: 58% AD group Screening sheet was used for 81% of the patients. 76% patients had all five screening measurements documented in their medical charts. Waist circumference: 18% Fasting glucose: 12%
Najim & Islam, 2013, UK (Najim & Islam, 2013)	Retrospective case note review	Basildon University Hospital Pharmacy, n = 65	Not reported	Not reported	Baseline: BP and pulse: 21.54% Weight: 10.77% Triglycerides: 6.15% HbA1c/Glucose: 29.23% Urea and electrolytes: 36.92% Liver function tests: 36.92% (48.25% had physical examination checked, 40% not checked and 11.75% not documented) Monitored six monthly for the first year Weight: 1.54% Triglycerides: 3.08% HbA1c/Glucose: 29.23% Urea and electrolytes: 9.23% Liver function tests: 10.77%
Butler et al., 2013, US (Butler et al., 2013)	Pre/during intervention review of charts	Acute inpatient psychiatry unit Preintervention n = 100	Not reported	Not reported	BP: 100% Weight: 100% Lipid Panel: 12% Fasting Blood Glucose: 39% Haemoglobin A1c: 7% Waist Circumference: 0%
Harrison et al., 2012, UK (Harrison et al., 2012)	Pre/post intervention audit	Acute adult psychiatric wards Baseline n = 85 • Ward A, 37 • Ward B, 48	Consultant psychiatrist and medical team	Not reported	Ward A BP: 83.7% Weight: 13.5% BMI: 4.7% Cholesterol: 40.5% Waist: 0% Prolactin: 10.8% Diabetes: 78.4% Abnormal Movements: 18.9% Ward B BP: 91.7% Weight: 12.5% BMI: 4.2% Cholesterol: 37.5% Waist: 0% Prolactin: 8.3% Diabetes: 58.3% Abnormal Movements: 14.6%
Coakley et al., 2012, US (Coakley et al., 2012)	Audit (Chart review)	Psychiatric hospital, n = 125	Not reported	Not reported	BP: 100% BMI: 100% HDL: 59.2% Triglycerides: 59.2% Fasting plasma glucose 71.2% Waist circumference: 60.8%
O’Callaghan et al., 2011, Ireland (O’Callaghan et al., 2011)	Pre/post intervention audit	General adult psychiatry—Outpatient clinic Initial audit n = 64	Nursing staff, psychiatrists and trainee doctors were responsible for monitoring	Not reported	Systolic BP 4.7% Diastolic BP 4.7% Weight 1.6% Height 0% HDL 12.5% Triglycerides 12.5% Serum fasting glucose 15.6% Waist circumference 1.6%
Bobes et al., 2011, Spain (Bobes et al., 2011)	Pre/post implementation study	Multiple—since targets psychiatrists not settings Psychiatrists n = 229 Patients n = 1193	Not reported	Not specified	Weight: 58.9% BMI: 32.8% Lipid profile: 69.6% Blood glucose: 70.6% Waist circumference: 19.2%
Thompson et al., 2011, Australia (Thompson et al., 2011)	Pre/post intervention audit	Public youth mental health service for those aged 15–25 years Pre- intervention n = 106	Interventions for metabolic problems offered by clinicians (case managers and psychiatrists) – roles and responsibilities not clearly described	Not reported	Pre intervention: Approximately 20% had minimum metabolic screening—defined as the completion of a full ‘set’ of metabolic measures including obesity measures (BMI or weight and height or waist-hip ratio); and metabolic blood tests (lipids and glucose) at some point within 6 months of being prescribed an antipsychotic Less than 10% had minimum metabolic monitoring—defined as the completion of full baseline measures including both obesity measure (BMI/waist hip ratio/or weight) and metabolic blood tests plus the completion of full measures at between 1–6 months following initiation of antipsychotic medication (or 1–6 months after baseline)
Batscha et al., 2010, US (Batscha et al., 2010)	Audit (Chart review)	Inpatient, specialty metabolic clinic, and outpatient n = 40 • Inpatient, 12 • Specialty metabolic clinic, 9 • Outpatient, 19	Inpatient setting: Monitoring conducted by physician or nurses based on orders	Not reported	Inpatient BP: 100% Weight: 100% Blood lipids: 8.3% Blood glucose: 58.3% Waist circumference: 8.3% Outpatient BP: 36.8% Weight: 36.8% Blood glucose: 10.5% Blood lipids: 5.3% Waist circumference: 0% Metabolic clinic BP: 77.8% Weight: 77.8% Blood lipids: 0% Blood glucose: 77.8% Waist circumference: 77.8%
Holt et al., 2010, UK (Holt et al., 2010)	Prevalence study	Department of Psychiatry Inpatients n = 50 Outpatients n = 50	Not reported	Not reported	Outpatient BP: 4% Weight: 0% Lipid profile: 8% Fasting glucose: 6% Any glucose: 14% Waist circumference: 0% Inpatient BP: 60% Weight: 6% Lipid profile: 10% Fasting glucose: 6% Any glucose: 18% Waist circumference: 0%
Marsay & Szabo, 2010, South Africa (Marsay & Szabo, 2010)	Retrospective case note review	Outpatient department of a specialist psychiatric hospital Patients prescribed olanzapine Commenced olanzapine as outpatients n = 16 Commenced as inpatients n = 23	Not reported	Not reported	See Table 2
Gumber et al., 2010, UK (Gumber et al., 2010)	Audit	Metabolic clinic Patients on atypical antipsychotics Initial audit (May 2006 and December 2007) n = 54 (48 attended baseline appointments) Repeat-audit- (December 2007 and January 2009) n = 123	Junior specialty trainees and General Practitioners (GPs)	Metabolic parameters monitored by junior specialty trainees, and abnormal results were sent to GPs. At the end of 1 year, the responsibility of annual monitoring was passed on to GPs.	BP: 100% BMI: 99% Cholesterol: 94% HDL: 74% Triglycerides: 94% Plasma glucose fasting or random: 79% Waist circumference: 99%
Nguyen et al., 2009, Australia (Nguyen et al., 2009)	Audit	Acute wards of public psychiatric hospitals, n = 93	Not reported	Not reported	Weight: 65% Height: 61% BMI: 0% Cholesterol: 7.5% HDL: 1.1% LDL: 1.1% Triglycerides: 7.5% Random blood sugar levels: 31% Postprandial blood sugar levels: 3.2% HbA1c: 2.2% Girth: 0%
Barnes et al., 2008, UK (Barnes et al., 2008)	Pre/post intervention audit	Secondary care mental health services Baseline audit n = 1966	Not reported	Not reported	BP: 26% BMI (or other obesity measure): 17% Plasma lipids: 22% Plasma glucose (or HbA1c): 28%
Verdoux et al., 2008, France (Verdoux et al., 2008)	Survey	Hospitals Psychiatrists n = 43	Assessment can be performed by psychiatrist, nurse, and general practitioner. The assessments were most often performed by a psychiatrist. The general practitioners were rarely implicated in the baseline screening. The other health professionals were a cardiologist, a nurse and the staff of the psychiatric emergency department.	Not reported	BP: 72.9% Weight: 61.5% Height: 56.7% BMI: 28.1% Total cholesterol: 61.1% HDL-cholesterol: 46.9% LDL-cholesterol: 46.9% Triglycerides: 61.1% Plasma glucose: 66.7% Waist circumference: 10.3% All measurements: 4.7% No measurement: 27.9%
Barnes et al., 2007, UK (Barnes et al., 2007)	Audit	All hospital trusts and private health care organisations that provide specialist mental health services Participating assertive outreach teams n = 53 Patient n = 1966	Patients were treated by the Assertive Outreach Teams* * Not specified	Patients were treated by Assertive outreach teams, however the specific monitoring process was not reported	BP: 26% BMI/or other obesity measures: 17% Plasma lipids: 22% Plasma glucose (or HbA1c): 28% Results for all 4 measures were documented in the case notes for 11% of patients overall, although the figure varied across the 21 services from 0 to 40%
Kilbourne et al., 2007, US (Kilbourne et al., 2007)	Population-based retrospective study	Veterans Administration Medical Centre Number of patients who were taking SGAs n = 252	Not reported	Not reported	Lipids o Total cholesterol: 49.6% o Triglycerides: 49.2% Serum fasting glucose level: 68.7% Recommended cardiovascular risk factor laboratory tests in less than 6 months: 50%
Feeney & Mooney, 2005, Ireland (Feeney & Mooney, 2005)	Audit	Rural Public Mental Health Services, n = 80	Not reported	Not reported	See Table 2

*HDL* = High Density Lipoprotein, *LDL* = Low Density Lipoprotein, *TG* = Triglyceride, *HbA1c *= haemoglobin A1c, *BMI* = Body Mass Index, *BP* = Blood Pressure

The studies covered a variety of settings, including hospitals (n = 14) (Coakley et al., 2012; Deuschle et al., 2013; Fontaine et al., 2022; Harrison et al., 2012; Holt et al., 2010; Hor et al., 2016; Kelly et al., 2022; Kjeldsen et al., 2013; Lydon et al., 2021; Najim & Islam, 2013; Nguyen et al., 2009; Shamima Saloojee et al., 2014a, 2014b; Tan et al., 2022; Verdoux et al., 2008), outpatient clinics (n = 5) (Kioko et al., 2016; Knudsen et al., 2022; Marsay & Szabo, 2010; O’Callaghan et al., 2011; Pereira et al., 2019), inpatients (n = 4) (Butler et al., 2013; Mwebe et al., 2020; Tatreau et al., 2016; Viglione & Short, 2021), secondary care settings (n = 2) (Barnes et al., 2008; Ross et al., 2018), primary care (n = 3) (Ali et al., 2023; Cotes et al., 2015; Stephenson et al., 2023), rural mental health services (n = 2) (Bomboy et al., 2021; Feeney & Mooney, 2005), general practices (n = 2) (Keenan et al., 2020; Lau et al., 2019), Veteran Administration Medical Centres (n = 2) (Kilbourne et al., 2007; Mittal et al., 2014), and one each from a tertiary care institution (Poojari et al., 2020), UK member trusts and healthcare services (Barnes et al., 2020), a metabolic clinic (Gumber et al., 2010) and a youth mental health service (Thompson et al., 2011). Six studies were conducted in multiple settings (Barnes et al., 2007, 2015; Batscha et al., 2010; Bobes et al., 2011; O’Brien & Abraham, 2021; Pearsall et al., 2019).

### Roles and Responsibilities

The healthcare professionals involved in metabolic monitoring (such as ordering blood tests, screening or documenting) included prescribers (Bomboy et al., 2021), junior medical officers (Gumber et al., 2010; Viglione & Short, 2021), family physicians (Ross et al., 2018; Stephenson et al., 2023; Tatreau et al., 2016), attending physicians (Ross et al., 2018) and psychiatrists (Deuschle et al., 2013; Tatreau et al., 2016; Verdoux et al., 2008). Others included nursing staff (Lydon et al., 2021), clinic staff (Bomboy et al., 2021), physiotherapists (Kjeldsen et al., 2013), healthcare assistants (Hor et al., 2016) and clinical pharmacists (Kjeldsen et al., 2013). Medical doctors (such as junior medical officers, physicians and psychiatrists) were often described as being involved in patient assessments, ordering blood tests and providing clinical interventions. Other health professionals, such as nurses, were often involved in conducting screening and/or physical assessments (Bomboy et al., 2021; Fontaine et al., 2022; Hor et al., 2016; Kjeldsen et al., 2013; Mwebe et al., 2020; O’Callaghan et al., 2011; Viglione & Short, 2021), while pharmacists had limited involvement in patient metabolic monitoring. One study described the role of clinical pharmacists in providing education and medication reviews during weekly outreach visits (Kjeldsen et al., 2013).

Most studies did not stipulate the specific roles and responsibilities of healthcare professionals involved in the metabolic monitoring process. For instance, a multiyear audit mentioned the role of the mental health team (specific health professionals not specified) in the assessment and recording of metabolic parameters for one year (2012) but not for subsequent years (Barnes et al., 2015). Another study alluded to psychiatric clinical staff (including resident physicians) having a role in metabolic monitoring in relation to education for participants (Ross et al., 2018). Although the study described patient care provided by consultants, psychiatric residents, nurses, occupational therapists and social workers, there was no description of the role and responsibilities of the clinical team.

### Procedure and Reported Metabolic Monitoring Practice

Seven studies reported on metabolic monitoring processes (Table 1) (Bomboy et al., 2021; Deuschle et al., 2013; Gumber et al., 2010; Kjeldsen et al., 2013; Lydon et al., 2021; Ross et al., 2018; Tatreau et al., 2016). Doctors, including psychiatrists and junior speciality trainees, were reported to be involved in the monitoring and documenting of metabolic parameters (Deuschle et al., 2013; Ross et al., 2018). Gumber and colleagues reported on a metabolic clinic managed by junior speciality trainees involving the monitoring of metabolic parameters at baseline and at 3, 6 and 12 months (Gumber et al., 2010). Any abnormal results were communicated to the GPs for their attention and appropriate intervention for the first year only. Thereafter, the responsibility for annual monitoring was transitioned to the GPs (Gumber et al., 2010). Similarly, several studies had described a screening process carried out by clinic staff, with participants assessed and reviewed by the prescriber (Bomboy et al., 2021; Tatreau et al., 2016). Blood samples were drawn onsite and subsequently transported to an offsite laboratory for processing, while the clinic staff were responsible for recording the results on the metabolic form (Bomboy et al., 2021). In all, three studies reported that blood test results were collected onsite and sent to a laboratory for assessment (Bomboy et al., 2021; Kjeldsen et al., 2013; Lydon et al., 2021). None of the studies stipulated whether metabolic monitoring was a mandated part of patient care, although three studies reported this as part of routine care (Batscha et al., 2010; Butler et al., 2013; Fontaine et al., 2022).

### Monitoring of Metabolic Parameters

**Fig. 2:**
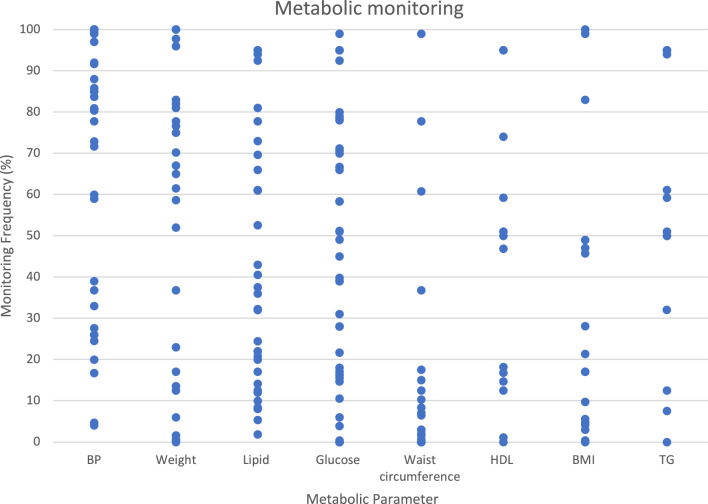
The type and frequency of metabolic monitoring for different metabolic parameters reported by the 29 included studies.*Studies that reported the frequency of metabolic monitoring over multiple years or where the prevalence was not reported were not included in this figure. Areas of overlap indicate that two or more studies reported similar monitoring rates

Several studies had measured various parameters (Table 1). Blood glucose levels varied between studies and were often reported as fasting glucose, HbA1c, and postprandial and random blood sugar levels. Figure 2 illustrates both the types and frequency of metabolic parameters measured in the included studies.

A total of 29 studies reported the frequency of metabolic monitoring for specific parameters, with some studies reporting these data for multiple cohorts (Ali et al., 2023; Barnes et al., 2020; Barnes et al., 2007; Barnes et al., 2008; Batscha et al., 2010; Bobes et al., 2011; Butler et al., 2013; Coakley et al., 2012; Cotes et al., 2015; Deuschle et al., 2013; Fontaine et al., 2022; Gumber et al., 2010; Harrison et al., 2012; Holt et al., 2010; Keenan et al., 2020; Kelly et al., 2022; Knudsen et al., 2022; Lau et al., 2019; Lydon et al., 2021; Nguyen et al., 2009; O’Brien & Abraham, 2021; O’Callaghan et al., 2011; Ross et al., 2018; Shamima Saloojee et al., 2014a, 2014b; Stephenson et al., 2023; Tan et al., 2022; Tatreau et al., 2016; Verdoux et al., 2008; Viglione & Short, 2021). Monitoring rates varied considerably across the 29 studies. Waist circumference (46%) and BMI (46%) were often poorly monitored, with nearly half of the studies reporting monitoring rates less than 20%. Lipids were also infrequently monitored, with many studies (54%) reporting rates of 40% or less. Just over half (55%) of the studies reported BP monitoring rates exceeding 70%, but blood glucose monitoring exhibited significant variation across studies, lacking a discernible pattern or cluster (Fig. 2).

Even though the types of metabolic parameters monitored varied between the included studies, most of the studies did not report on all five metabolic parameters. Waist circumference, HDL, BMI and TG levels were not as frequently monitored in these studies. Najim and colleagues suggested that there was inadequate metabolic monitoring and reported that less than half (48.25%) had a baseline physical health examination (Najim & Islam, 2013). Similarly, Kioko and colleagues reported that only 22% of patients had appropriate laboratory tests available (Kioko et al., 2016). In contrast, a cross-sectional retrospective review of records for people with SMIs revealed that most (83.1%) had evidence of routine blood monitoring (including glucose, cholesterol, HbA1c and TG) within the preceding two years (Pearsall et al., 2019).

Five studies explored the frequency of metabolic monitoring (retrospectively) at different time points (Feeney & Mooney, 2005; Marsay & Szabo, 2010; Mwebe et al., 2020; Najim & Islam, 2013; Poojari et al., 2020). Of these, four that examined the frequency of routine metabolic monitoring in the same cohort reported a reduction in monitoring rates for all metabolic parameters over time (Table 2) (Feeney & Mooney, 2005; Marsay & Szabo, 2010; Najim & Islam, 2013; Poojari et al., 2020). One study reported higher metabolic monitoring rates for all parameters (BMI, BP, glucose, lipid and electrocardiogram monitoring) except waist circumference for patients at three months after admission than at baseline (Mwebe et al., 2020).

**Table 2:** Frequency of routine metabolic monitoring over time

Authors	Parameters	Baseline (%)		3-month (%)		Annually (%)
Poojari et al., 2020 (Poojari et al., 2020)	Weight	Baseline: 60		7.6		79.4
		1 month: 14.9		Quarterly: 9.8		
		2 months: 2.5				
		3 months: 7.6				
	Waist circumference	1		Not reported		0
	Blood pressure	99.7		14.6		72.7
	Glucose	47		12.4		72.7
	Lipid	39.7		5.1		27.9
Mwebe, Volante, and Weaver 2020 (Mwebe et al., 2020)	BMI	74		98		
	Waist circumference	Not reported		5		
	Blood pressure	90		100		
	Glucose	77		84		
	Lipid	59		79		
Najim & Islam, 2013 (Najim & Islam, 2013)	Weight	Baseline		6-month		
		10.77		1.54		
	Blood pressure and pulse	21.54		Not reported		
	Glucose	29.23		29.23		
	TG	6.15		3.08		
Feeney & Mooney, 2005 (Feeney & Mooney, 2005)	Weight	Baseline		Ongoing monitoring*		
		40		15		
	BMI	0		0		
	Blood pressure	45		17.5		
	Glucose	21.3		22.5		
	HbA1c	3.8		8.8		
	Lipids	6.3		17.5		
Marsay & Szabo, 2010 (Marsay & Szabo, 2010) (Outpatient)	Weight	Baseline	1 month	2 months	3 months	4 months
		6	6	6	13	25
	Blood pressure	6	6	6	6	6
	Glucose	19	0	0	0	0
	Lipids	13	0	0	0	0
	Cholesterol	6	0	0	0	0
Marsay & Szabo, 2010 (Marsay & Szabo, 2010) (Inpatient)	Weight	73.91	17.39	8.7	0	4.35
	Blood pressure	100	82.61	52.17	21.74	17.39
	Glucose	13.04	4.35	0	0	4.35
	Lipids	17.39	4.35	4.35	0	4.35
	Cholesterol	8.7	4.35	0	0	4.35

^*^Evidence of monitoring test being carried out in the past year

Batscha and colleagues compared the frequency of metabolic monitoring across three different settings: inpatient, outpatient and metabolic clinic (Batscha et al., 2010). The authors reported that weight (p < 0.001) and BP (p < 0.001) were most frequently measured in an inpatient setting, while glucose (p < 0.001) and waist circumference (p < 0.001) were more regularly measured in the outpatient setting. Furthermore, waist circumference was more likely to be monitored in the metabolic clinic than in the inpatient unit (p < 0.001)(Batscha et al., 2010). Similarly, Marsay and colleagues reported that the metabolic parameters of the inpatient cohort were monitored more often than those of their outpatient counterparts (Marsay & Szabo, 2010).

## Discussion

Metabolic monitoring for SMI appears suboptimal, and metabolic conditions such as CVD are often undiagnosed and untreated (Heiberg et al., 2019). Research to date has explored barriers to metabolic monitoring in the SMI, highlighting barriers at the individual, organisational, and systems levels (Ali et al., 2020; Cunningham et al., 2018). However, additional research is needed to address this gap (Solmi et al., 2021). This review explored the nuances of metabolic monitoring, offering an overview of current metabolic monitoring practices for SMI. Specifically, regarding the roles and responsibilities of healthcare professionals, specific metabolic parameters were monitored, as were the types of approaches and methodologies used in routine metabolic monitoring. Our findings have highlighted several gaps in current practice, including a lack of standardised metabolic monitoring procedures and processes and suboptimal metabolic monitoring rates for SMIs taking antipsychotics (Ali et al., 2021, 2023).

The uncertainty surrounding the specific roles and responsibilities of health professionals involved in care for SMIs can impede routine physical health monitoring (Mitchell et al., 2012; Poojari et al., 2023; Roughead et al., 2017). Clarity around who is involved in particular aspects of care is pivotal, particularly for SMI, as care is often delivered by a multidisciplinary team (Ali et al., 2020; Aouira et al., 2022; Roughead et al., 2017). The majority of studies identified in this review did not describe the specific roles and responsibilities of various healthcare professionals on clinical care teams. Although studies have listed the specific healthcare professional(s) involved in the care of SMI, often, their roles and responsibilities have been poorly defined (Barnes et al., 2007, 2015, 2020; Batscha et al., 2010; Cotes et al., 2015; Fontaine et al., 2022; Gumber et al., 2010; Harrison et al., 2012; Hor et al., 2016; Mwebe et al., 2020; O’Callaghan et al., 2011; Ross et al., 2018; Stephenson et al., 2023; Thompson et al., 2011). Furthermore, none of the studies reported whether metabolic monitoring was mandated as part of routine care in the specific study setting. Most of the studies identified in this review involved interventional methods and therefore may not necessarily involve reporting on metabolic monitoring practices in a specific setting. In addition, word count limits imposed by journals could deter authors from reporting detailed metabolic screening and/or monitoring practices.

Only seven of the included studies reported the process involved in metabolic monitoring for SMI in a particular setting (Bomboy et al., 2021; Deuschle et al., 2013; Gumber et al., 2010; Kjeldsen et al., 2013; Lydon et al., 2021; Ross et al., 2018; Tatreau et al., 2016). Specific procedures and processes, such as whether blood samples were analysed onsite or off-site, were infrequently reported. Presumably, studies conducted within a hospital setting would have access to onsite pathology facilities, while primary care settings, such as general practices, would rely on offsite laboratories for analysis. However, this may not always be the case, such as in rural areas (Blattner et al., 2019). The absence of an onsite laboratory facility and services would reduce the accessibility and convenience of conducting and obtaining timely blood test results, therefore affecting metabolic monitoring rates. In addition, doctors had previously suggested that the lack of a notification system that alerts patients when blood test results are ready and/or when further action is required is a barrier to optimal metabolic monitoring (Aouira et al., 2022). Further attention is warranted to support healthcare delivery for vulnerable populations, including SMIs.

Aligned with the literature, our findings highlighted suboptimal metabolic monitoring for SMI (Ali et al., 2021, 2023; Mitchell et al., 2012). Among the studies included in this review, Lydon and colleagues recorded the highest rates of metabolic monitoring for patients with SMI who were taking clozapine (Lydon et al., 2021). This was not unexpected given the serious side effects and potential fatalities related to clozapine use (Kar et al., 2016). The prescribing and dispensing of clozapine are also associated with mandatory monitoring requirements in most countries, including Australia and the UK (Medicinewise, 2022; NHS Trust, 2023). However, monitoring of other SGAs requires further attention; for example, Keenan and colleagues reported that none of their study participants (n = 117) were fully monitored according to the RANCZP monitoring guidelines (Keenan et al., 2020). Previous reports had identified a number of barriers to routine metabolic monitoring, including patient-related barriers such as the perception of laboratory testing as aversive and intrusive (O’Brien & Abraham, 2021) and low levels of health literacy or awareness (S. Saloojee et al., 2014a, 2014b). The clinician-related factors identified included insufficient time (Kioko et al., 2016) and/or lack of reimbursement (Batscha et al., 2010). Future research initiatives to improve metabolic monitoring for SMI should consider previously identified, in addition to any other barriers that may be relevant to the particular setting or demographic. It is worth noting that given the variability across the studies reported (e.g. settings and countries), the perceived suboptimal monitoring in some contexts may be considered acceptable in others.

Our review revealed that parameters such as waist circumference and BMI are often poorly measured (Fig. 2). Barriers to frequent monitoring of these parameters, particularly waist circumference, have been previously explored in the literature (Hor et al., 2016; Mitchell et al., 2013; Verdoux et al., 2010). One study attributed low waist circumference monitoring rates to patients’ preferences for healthcare professionals of the same sex, who may not always be readily available (Hor et al., 2016). Verdoux and colleagues also cited sex differences as a potential barrier, noting that psychiatrists might be reluctant to perform physical examinations, especially for female patients, as such examinations can be “*construed as invasive (such as waist measurement)* (Verdoux et al., 2010).” There is a need to improve waist circumference monitoring rates in the SMI, particularly because it has been shown to be a useful predictor of MetSyn, with high rates of sensitivity and specificity (Mitchell et al., 2013).

The observed variation in metabolic monitoring rates between settings should be noted by clinicians and policymakers. At a practice level, clinicians should recognise that patients may not receive regular metabolic monitoring as suggested by existing guidelines. Consideration for the need and potential value of metabolic monitoring should be considered at patient interaction and a review should be initiated if deemed appropriate. Policymakers (e.g.in hospitals) should also facilitate frequent metabolic monitoring within their local settings. This can be achieved through local quality improvement efforts and implementation of mandatory clinical guidelines and/or policies at an organisational level, which can influence clinical practice. Clinical guidelines and/or policies must be tailored towards the needs of the particular site and consider other factors including staffing and resourcing requirements.

### Strengths and limitations

A strength of this review was the methodologically rigorous search of published and grey literature in line with JBI recommendations. The search strategy, including its translation across databases, was guided by an academic librarian, and reviewed by the research team to ensure that a comprehensive and relevant search was conducted. However, this review included only English-language studies and was mostly based in the UK and US and from tertiary settings (hospital, inpatient and outpatient services). This skewed representation may reduce the generalisability of the present findings to other countries and settings.

### Future Research

Despite the publication of practice guidelines, these findings suggest that metabolic monitoring in practice remains suboptimal, with procedures and processes varying between settings. As most of the studies included in this review did not report on the metabolic monitoring processes at the local site, we were unable to review and identify factors that influenced metabolic monitoring rates. Future research should consider the need to explore local metabolic monitoring practices, particularly to identify the barriers and facilitators relevant to the specific setting.

While this review echoes findings from previous research (Mitchell et al., 2012), it also highlighted the observed variation in metabolic monitoring. Future studies should also consider exploring the temporal trends to compare the prevalence of metabolic monitoring over the years.

Future practice guidelines should consider recommending and specifying the roles and responsibilities of healthcare professionals in metabolic monitoring for SMI. Researchers should clearly describe the local metabolic monitoring practices and/or procedures where possible to allow their findings to be appropriately interpreted and to facilitate the implementation of interventions in other settings where practices and/or procedures may differ. There is a need for ongoing research, particularly in the community setting, to promote increased accessibility to metabolic monitoring for SMI.

## Conclusion

This scoping review mapped out the nuances of metabolic monitoring in practice. The most common settings, types of parameters measured, and health professionals involved in metabolic monitoring were summarised. The scoping review indicates that no streamlined approach toward metabolic monitoring currently exists, with variations observed between different settings and countries. This information highlights the need for a more systematic approach to metabolic monitoring for SGA patients with SMI. Our findings also highlight the need to clearly stipulate and define the roles and responsibilities of all health professionals involved in metabolic monitoring for SMI. Clinicians should be aware of the existing variations in metabolic monitoring and policymakers should consider the need to facilitate metabolic monitoring at an organisational level, taking into consideration existing resources and the specific needs of the organisation.

## Supplementary Information

Below is the link to the electronic supplementary material.

Supplementary file1 (DOCX 15 kb)

Supplementary file2 (PDF 553 kb)
