# Supplementary material for: Metabolic Monitoring for Adults Living with a Serious Mental Illness on a Second-Generation Antipsychotic Agent: A Scoping Review
Source: Adm Policy Ment Health. 2024 Aug 17;52(2):289–317. doi: 10.1007/s10488-024-01408-9 (PMC11903528; doi:10.1007/s10488-024-01408-9)
Supplement: Supplementary file 1 — Supplementary file1 (DOCX 15 kb) [file 10488_2024_1408_MOESM1_ESM.docx]

**Supplementary Information 1: Search strategy for Medline**

Mental Disorders/

exp "Bipolar and Related Disorders"/

exp Mood disorders/

Mania/

exp "Schizophrenia Spectrum and Other Psychotic Disorders"/

((mental* or neurodevelopment* or behaviour or behavior or psychologic* or neuropsychiatr* or neuro psychiatr* or psychiatr* or psychopatholog* or psycho patholog* or psychic or psychotic or psychos*) adj5 (confusion or defect or change or symptom* or insufficien* or abnormal* or episode* or disorder* or condition* or ill* or feature* or spectrum or state or disease or disturb* or confusion or defect* or diagnos*)).ti,ab,kf.

(Insan* or bipolar or bi polar or schizophreni* or schizo phreni* or schizo affective or schizoaffective or paranoi* or delusion or mania or manic or bipolar or bi polar or cyclothymi* or psycho* or mood or hallucin* or hypomania or hypo mania).ti,ab,kf.

((depress* or cyclothymi* or affective or mood) adj2 (psycho* or disorder* or disturbance or illness*)).ti,ab,kf.

(schizophreni* or schizo phreni* or dementia praecox or depress* or melancholia or paraphrenia* or para phrenia).ti,ab,kf.

((schizophreni* or schizo phreni* or affective) adj2 (psychos* or incipient or borderline or latent or disorder or pseudoneurotic or pseudo neurotic or pseudopsychopathic or pseudo psychopathic)).ti,ab,kf.

Mentally ill persons/

((mental or psychiatr*) adj2 (patient* or diagnosis* or state or person* or people)).ti,ab,kf.

1 or 2 or 3 or 4 or 5 or 6 or 7 or 8 or 9 or 10 or 11 or 12

Tranquilizing agents/

exp antimanic agents/

exp antipsychotic agents/

(antipsycho* or anti psycho* or antimani* or anti mani* or neuroleptic* or tranquiliz* or tranquilis* or anti schizophren* or antischizophren* or psychotropic).ti,ab,kf.

(Droperidol or zuclopenthixol or clozapine or chlorpromazine or flupentixol or haloperidol or iloperidone or lumateperone or lurasidone or olanzapine or paliperidone or quetiapine or risperidone or ziprasidone or aripiprazole or trifluoperazine or cariprazine or pimavanserin or pericyazine or periciazine or amisulpride or asenapine or brexipiprazole).ti,ab,kf.

14 or 15 or 16 or 17 or 18

Metabolic diseases/

exp Heart Disease Risk Factors/

exp Diabetes Mellitus/

exp overweight/

body mass index/

Hyperglycemia/

hypertension/

exp insulin resistance/

blood pressure/

Lipids/

triglycerides/

cardiovascular diseases/

exp body size/

(body size or waist circumference or body composition or body weight* or body height or body measure* or anthropometr* or waist size).ti,ab,kf.

(insulin resistance or metabolic or cardiometabolic or cardio metabolic or dysmetabolic or dys metabolic or Reaven or cardiovascular or cardio vascular or syndrome X).ti,ab,kf.

(diabetes or glucose or hyperglycemi* or hyper glycemi* or hyperglycaemi* or hyper glycaemi* or HbA1c or hemoglobin A1c or hyperinsulin* or hyper insulin*).ti,ab,kf.

(obesity or overweight or over weight or weight or body mass index or BMI).ti,ab,kf.

(dyslipidemia or dys lipidemia or triglyceride* or lipid* or cholesterol or cholesterolemia or hyperlipidemia or hyper lipidemia).ti,ab,kf.

(hypertension or hyper tension or blood pressure or BP or diastol* or systol*).ti,ab,kf.

20 or 21 or 22 or 23 or 24 or 25 or 26 or 27 or 28 or 29 or 30 or 31 or 32 or 33 or 34 or 35 or 36 or 37 or 38

Mass Screening/

Monitoring, Physiologic/

((screen* or measur* or examin* or monitor* or assess* or evaluat* or manage* or intervention or intervene or improve) adj5 (physical or health or physiologic* or routine or practice* or patient or metabolic or cardiometabolic or cardio metabolic)).ti,ab,kf.

40 or 41 or 42

13 and 19 and 39 and 43
